# A Retrospective Clinico-Pathologic Study of 35 Dogs with Urethral Transitional Cell Carcinoma Undergoing Treatment

**DOI:** 10.3390/ani13142395

**Published:** 2023-07-24

**Authors:** Giulia Ghisoni, Armando Foglia, Silvia Sabattini, Chiara Agnoli, Francesco Dondi, Simone Perfetti, Laura Marconato

**Affiliations:** Department of Veterinary Medical Sciences, Alma Mater Studiorum University of Bologna, 40064 Ozzano dell’Emilia, BO, Italy; giulia.ghisoni@unibo.it (G.G.); armando.foglia2@unibo.it (A.F.); silvia.sabattini@unibo.it (S.S.); chiara.agnoli2@unibo.it (C.A.); f.dondi@unibo.it (F.D.); simone.perfetti4@unibo.it (S.P.)

**Keywords:** urethra, urothelial carcinoma, canine, prognosis, urethral obstruction, urinary tract infection

## Abstract

**Simple Summary:**

Transitional cell carcinoma is the most common tumor of the urinary tract in dogs. Its uncommon occurrence in the urethra presents numerous challenges, and the clinician’s decision-making process is complicated by the absence of reliable prognostic factors. In this retrospective study, we examined data from 35 dogs with histologically confirmed urethral carcinoma, including staging work-up, medical treatment, and outcome information, in order to determine the impact of various variables on disease progression and survival. Our analysis revealed that urethral obstruction and urinary tract infection at the time of admission were adverse prognostic factors in dogs with urethral carcinoma. Therefore, a multimodal therapeutic approach should be considered to enhance the outcomes in these patients.

**Abstract:**

Chemotherapy and cyclooxygenase inhibitors (COXi) are primary treatments for canine urethral transitional cell carcinoma (uTCC), a tumor known for its aggressiveness and poor prognosis. This retrospective study aimed to evaluate the clinico-pathological characteristics, treatment modalities, and prognostic factors of 35 dogs with confirmed uTCC that received chemotherapy and COXi. Upon admission, urethral obstruction (UO) and urinary tract infection (UTI) were observed in seven (20%) dogs each. Gemcitabine (*n* = 20; 57.1%) and vinblastine (*n* = 10; 28.6%) were commonly used as first-line therapies, with four dogs also receiving radiation therapy. Based on RECIST, one (2.9%) dog achieved complete remission, nine (25.7%) partial remission, 20 (57.14%) showed stable disease, and five (14.3%) progressed. Among dogs with UO, six (85.7%) showed resolution or improvement after the first chemotherapy dose. The median time to local progression was 171 days (range: 107–235), and the median survival time was 333 days (range: 158–508). Dogs with UO upon admission had a higher risk of local progression, while both UO and UTI were associated with an increased risk of overall disease progression and tumor-related death. Additionally, gemcitabine significantly improved metastatic control. This study identified UO and UTI as negative prognostic factors, highlighting the importance of a multimodal approach in managing uTCC.

## 1. Introduction

In dogs, transitional cell carcinoma (TCC) is the most common malignant tumor of the lower urinary tract, accounting for approximately 2% of all reported canine tumors [1,2]. TCC is suspected to have a multifactorial etiology, with risk factors including female sex, neuter status, obesity, and exposure to pesticides [3,4]. Primary urethral localization is uncommon, and it is mostly diagnosed in spayed and aged female dogs [3,4,5]. In a study involving a population of 102 dogs with bladder TCC, cancer was found to affect the urethra in 56% of dogs and the prostate in 29% of male dogs [6].

Early detection of urethral TCC (uTCC) is often challenging due to nonspecific clinical signs, including hematuria, stranguria, and pollakiuria [6]. The biologic behavior of uTCC is aggressive, primarily due to partial or complete tumor-related urethral obstruction (UO). At the time of diagnosis, metastases to regional lymph nodes and distant organs are reported in 24% to 39% of dogs [7]. 

Due to its location, uTCC is usually not amenable to surgical excision [8], and the efficacy and side effects of radiation therapy (RT) are not well documented [9,10]. Chemotherapy, either alone or in association with cyclooxygenase inhibitors (COXi) [11], remains the standard treatment for managing nonresectable or metastatic disease [3,12,13,14,15,16,17]. 

Urethral obstruction (UO) is the leading cause of death in dogs with uTCC, and several local palliative treatments have been described to restore urethral patency. These include ultrasound-guided cystoscopic laser ablation, placement of urethral stent or cystostomy tubes, and surgical diversion [18,19,20,21,22]. 

Overall, prognosis for dogs with uTCC is poor, primarily due to local progression [21]. According to one study, a median survival time of 121 days was reported [1].

There is limited published literature on primary uTCC, thus, the aim of this retrospective study was to review the clinico-pathologic features and treatment modalities for dogs with uTCC, possibly identifying prognostic factors.

## 2. Materials and Methods

### 2.1. Sample Population

The study did not fall within the application areas of the Italian Legislative Decree 26/2014 which governs the protection of animals used for scientific or educational purposes; therefore, ethical approval was waived for this study. Dogs were treated according to the current standards. All owners signed a written informed consent.

The medical record database of the Veterinary Hospital of the University of Bologna was searched to identify dogs diagnosed with uTCC between January 2012 and April 2022. To be considered as primary uTCC, the tumor should extensively involve the urethra (covering more than 75% of the urethral length) with most of the tumor volume located within the urethral lumen. Urinary bladder or prostate involvement was considered if at least one singular papillary lesion, attributable to the tumor, was detected at the level of the vesico-urethral junction, the trigone, and/or the prostate, using a combination of endoscopy and abdominal ultrasound or CT.

Inclusion criteria for the study were histologic confirmation of uTCC, completion of staging work-up (abdominal ultrasound associated with 3-view thoracic radiographs or computed tomography of the thorax and abdomen, complete blood count, serum biochemistry panel, urinalysis, and urine culture if indicated), administration of any chemotherapy, and availability of outcome data. Dogs with simultaneous involvement of the prostate and/or bladder were also included. Exclusion criteria encompassed cases where TCC was limited to the bladder, staging was incomplete, chemotherapy was not administered, or there was a loss of follow-up information.

For each dog included in the study, relevant information was extracted from the medical records, including signalment (breed, age, sex, weight), pertinent previous medical history, presenting clinical signs, duration of clinical signs, clinico-pathologic findings, staging diagnostics, surgical interventions, chemotherapy regimen, treatment-related complications, treatment response, type and date of progression, rescue treatment, and date and cause of death. 

In all cases, the diagnosis was confirmed through histopathology, and biopsies were obtained either via endoscopy or ultrasound-guided traumatic catheterization.

To assess treatment response, serial ultrasounds were conducted, and the Response Evaluation Criteria in Solid Tumors (RECIST) [23] system was employed. Side effects resulting from medical therapy were graded based on the Veterinary Cooperative Oncology Group (VCOG) criteria [24].

### 2.2. Statistical Analysis

Descriptive statistics were used in the analysis of dogs and tumor characteristics. When appropriate, data were tested for normality by use of the D’Agostino and Pearson omnibus normality test. Values were expressed as mean ± SD in the case of normal distribution, or as median with a range in the case of non-normal distribution.

Time to local progression (TTLP) and time to metastasis (TTM) were calculated from the date of treatment to the date of the first-documented local recurrence or metastasis, respectively. Time to progression (TTP) was defined as the time from initiation of treatment to the first occurrence of disease progression (either local or distant).

Tumor-specific survival (TSS) was defined as the interval between initiation of treatment and tumor-related death. Only dogs deceased for uTCC-related causes were considered as events.

Survival plots were generated according to the Kaplan–Meier product-limit method. Survival estimates were presented as medians with the corresponding 95% confidence intervals (95% CI). The influence of potential prognostic variables on tumor progression or uTCC-related death was investigated with Cox’s regression analysis.

Variables investigated for prognostic significance included age, sex, breed, weight, symptoms duration, azotemia, urinary tract infection (UTI) and/or UO at diagnosis, bladder and/or prostate involvement, metastasis at diagnosis, development of UTI and/or UO during treatment, and need for additional procedures (i.e., cystoscopic laser ablation). Data were analyzed by use of commercial software programs (SPSS Statistics v. 26, IBM, Somers, NY, and Prism v. 5.0, GraphPad, San Diego, CA, USA).

The level of significance was set at *p* < 0.05.

## 3. Results

### 3.1. Patient Data and Tumor Characteristics

A total of 35 dogs diagnosed with uTCC were included in the study. There were 27 (77.1%) females, of which 23 were spayed, and eight (22.9%) males, with five being neutered. The median age was 11 years (range, 6–15), and the median weight was 19 kg (range, 5–45). 

Represented breeds included mixed breed (*n* = 16, 45.7%), Labrador Retriever (*n* = 3, 8.6%), Schnauzer (*n* = 2, 5.7%), Maremma sheepdog (*n* = 2, 5.7%), German shepherd (*n* = 2, 5.7%), Jack Russel Terrier (*n* = 2, 5.7%), and one (2.9%) each of the following: Golden Retriever, Yorkshire Terrier, Shitzu, Samoyedo, Scottish Shepherd, Chow Chow, Italian Segugio, and Spinone. 

All dogs in the study were referred due to urologic symptoms such as stranguria, pollakiuria, and pigmenturia, with a median duration of symptoms of 90 days (range, 14–365). A significant clinical finding was urethral thickening, detected through digital rectal examination, which was reported in 18 (51.4%) dogs. Additionally, seven dogs (20%) presented with a large-sized and painful urinary bladder, accompanied by the inability to void voluntarily, resulting in a diagnosis of UO upon presentation.

At the time of diagnosis, urine cultures were performed in 30 out of 35 dogs (85.7%), and a positive result was documented in seven (23.3%) dogs. The identified pathogens were Escherichia coli (*n* = 3), Pseudomonas aeruginosa (*n* = 1), Proteus mirabilis (*n* = 1), and Staphylococcus pseudointermedius (*n* = 1). Six dogs (17.1%) were azotemic at the time of diagnosis, with a median creatinine level of 1.95 mg/dL (range: 0.9–3.9, reference interval (RI): 0.75–1.4), a median urea level of 76.5 mg/dL (range: 51–150, RI: 17–48), and a median phosphorus level of 6.2 mg/dL (range: 4.1–7.7, RI: 2.6–5.4). In these patients, the main hypothesized causes of azotemia were acute on chronic kidney disease associated with chronic/recurrent urinary tract obstruction.

The diagnosis was obtained by cystoscopy in 21 (60%) dogs and by ultrasound-guided traumatic catheterization in 14 (40%). 

Imaging technology adopted for staging included thoracic radiographs and abdominal ultrasound in 29 (82.9%) dogs and TBCT in the remaining six (17.1%). Three dogs on which TBCT was not performed also underwent radiographic bone survey, in addition to thoracic radiography and abdominal ultrasound.

The urethral location of the tumor was documented in all dogs, and concomitant involvement of the bladder or prostate was reported in 22 (62.9%) and two (5.71%) dogs, respectively. The median thickness of the urethral lesions, measured using ultrasonography or CT, was 9 mm (range, 4–23). The extent of the lesions was not consistently assessable due to the potential extension of the lesion within the pelvic canal, particularly in ultrasonographic examinations.

Fine needle aspiration of regional lymph nodes was not routinely conducted. Lymph nodes were sampled only if they appeared abnormal on ultrasonography or tomography. All regional lymph nodes identified as metastatic were enlarged at the time of diagnosis and were subsequently sampled during the staging work-up.

Based on clinical staging, seven (20%) dogs had metastatic iliac lymph nodes, three (8.6%) dogs had metastatic iliac and inguinal lymph nodes, one (2.9%) dog had iliac lymph nodes and lung metastasis, and one (2.9%) dog had pulmonary metastasis. Only nodal metastases were confirmed by cytologic evaluation. The overall metastatic rate at admission was 34.4%.

### 3.2. Treatment

All dogs included in the study had nonresectable uTCC.

Chemotherapy in combination with COXi (consisting of oral piroxicam administered at 0.3 mg/kg/day) served as the primary treatment for all dogs included in the study. 

Sixteen (45.7%) dogs received weekly intravenous gemcitabine (800 mg/m^2^) for a median of four cycles (range, 1–45). Ten (28.6%) dogs were treated with intravenous biweekly vinblastine (3 mg/m^2^) for a median of eight cycles (range, 2–30). Carboplatin (300 mg/m^2^, every 21-day) was used on two (5.7%) dogs for five and seven cycles, respectively. Two (5.7%) dogs received oral daily chlorambucil (4 mg/m^2^) and one (2.9%) dog received one dose of intravenous mitoxantrone (5 mg/m^2^).

Four (11.4%) dogs underwent chemo-radiotherapy, consisting of four cycles of weekly neoadjuvant gemcitabine, followed by RT (12 fractions of 3 Gy, each for a total dose of 36 Gy over a 3-week period). Two weeks after the end of RT, these dogs also received four cycles of intravenous carboplatin at the dose of 240 mg/m^2^, once every 21 days. 

Retrograde indwelling urethral catheterization using a Foley catheter was initially employed in all seven dogs with UO upon presentation prior to initiating chemotherapy, and was left in place for 4–7 days after chemotherapy administration. 

The most frequently observed chemotherapy-related toxicities were gastrointestinal (GI) in nature. Five (14.3%) dogs experienced grade 2 GI toxicities, while four (11.4%) dogs experienced grade 1 toxicities. Additionally, two (5.7%) dogs developed grade 1 neutropenia after receiving chemotherapy, one (2.9%) dog developed a grade 2 fever in the absence of neutropenia, and increased azotemia was reported in one (2.9%) patient. No patients required hospitalization for the management of these side effects.

During treatment, the decision to renew the urine culture test was based on clinical suspicion of UTI. This complication was documented in 15 out of 35 (42.9%) dogs. Overall, 21 dogs had at least one positive urine culture test. UTIs were treated based on therapeutic indications derived from antimicrobial susceptibility patterns and/or the International Society for Companion Animal Infectious Disease (ISCAID) guidelines [25].

### 3.3. Outcome

Response data was available for all dogs, as evaluated through monthly ultrasonographic re-evaluation. 

Based on RECIST criteria, one (2.9%) dog treated with chemo-radiotherapy achieved complete remission (CR). This dog had a sustained response, being alive after 1215 days, showing no signs of local or distant progression. Nine (25.7%) dogs achieved partial remission (PR) following treatment with gemcitabine (*n* = 8) or vinblastine (*n* = 1), with a median TSS of 294 days (range, 91–637). Stable disease (SD) was observed as the best response in 20 (57.14%) dogs, with a median TSS of 209 days (95% CI, 78–340). Drugs administered in these 20 dogs included vinblastine (*n* = 9), gemcitabine (*n* = 6), carboplatin (*n* = 2), chlorambucil (*n* = 2), and mitoxantrone (*n* = 1). Five (14.3%) dogs developed progressive disease (PD) following one to four cycles of gemcitabine (*n* = 4) and chemo-radiotherapy (*n* = 1), with an overall median TSS of 64 days (range, 14–107). 

In addition to the dog that achieved CR, two of the four irradiated dogs attained PR with respective TTLP of 487 and 162 days; one progressed 21 days after treatment.

The overall response rate observed was 28.6%.

Twenty-four (68.6%) dogs experienced local progression and nine (25.7%) dogs developed distant progression. New metastatic sites included iliac lymph nodes (*n* = 3), sacral lymph nodes (*n* = 2), lung (*n* = 2), spleen (*n* = 2), liver (*n* = 1), inguinal lymph node (*n* = 1), vertebrae (*n* = 1), rib (*n* = 1), and skin (*n* = 1).

Overall, median TTLP was 171 days (95% CI, 107–235), while the median TTM was not reached. The median TTP was 162 days (95% CI, 79–245).

At the end of the study, 27 (77.1%) dogs had died due to tumor-related causes. The median TSS was 333 days (95% CI, 158–508), with 1- and 2-year survival rates of 29% and 7%, respectively. One dog had died due to TCC-unrelated causes (histologically confirmed meningioma) after 434 days. The remaining seven dogs were alive at the end of the study, with a median follow-up time of 289 days (range, 47–1215).

Among the seven dogs with UO at diagnosis, initially treated with transurethral catheterization, six (85.7%) experienced resolution of the obstruction after receiving the first dose of chemotherapy, consisting of vinblastine (*n* = 5) or gemcitabine (*n* = 1). The remaining patient received chemo-radiotherapy, but disease progression occurred within 21 days after the first cycle of carboplatin. 

During first-line chemotherapy, 13 (37.1%) patients developed UO, and in five (38.5%) of these dogs, additional procedures were performed. These included endoscopic-assisted laser debulking (*n* = 3), cystostomy tube placement (*n* = 1), and catheterization followed by a switch to a rescue chemotherapy protocol (*n* = 1).

Following documentation of PD, 20 (57.14%) dogs received rescue treatments, consisting of vinblastine (*n* = 6), carboplatin (*n* = 4), a combination of vinblastine and toceranib (*n* = 4), mitoxantrone (*n* = 2), gemcitabine (*n* = 1), chlorambucil (*n* = 1), or metronomic chemotherapy (*n* = 1). 

### 3.4. Risk Factor Analysis

At univariable analysis, variables associated with an increased risk of local progression included UO at diagnosis and need for additional procedures. Variables associated with an increased risk of distant progression included pure breed and medical treatment other than gemcitabine. Variables associated with an increased risk of overall tumor progression included UTI and UO at diagnosis (Table 1). Variables associated with an increased risk of tumor-related death included body weight > 19 kg and UO and UTI at presentation (Table 1).

In multivariable analysis, UO at diagnosis retained prognostic significance for local progression and medical treatment other than gemcitabine retained prognostic significance for distant progression, whereas UO and UTI at diagnosis were significant for both overall progression and tumor-related death (Table 2).

## 4. Discussion

Despite TCC being the most common malignant tumor in the canine urethra, the literature addressing this is sparse and prognostic factors are poorly characterized.

In the current retrospective study, 35 dogs with uTCC undergoing medical treatment were included. In most cases (23 of 27; 85%), death was due to local tumor progression, however, metastatic disease was reported in a remarkable proportion of cases (18 of 35; 51%). UO at admission was significantly (*p* = 0.008) associated with local progression, whereas gemcitabine administration was significantly (*p* = 0.047) associated with a decreased risk of distant progression. Overall, UO and UTI were identified as independent negative prognostic factors for both progression and tumor-related death.

UO and UTI are commonly reported as comorbidities in cases of urinary tract TCC [5]. Since UO often occurs prior to the development of metastasis, palliative treatments aimed at rapidly restoring urethral patency are frequently recommended to improve outcome [21]. In the present study, all dogs with UO received transurethral catheterization as the initial management approach before starting chemotherapy. Six out of seven dogs presenting with UO were successfully treated with vinblastine as a first-line chemotherapeutic agent, which restored urethral patency after the first dose without requiring additional interventional procedures. Contrastingly, endoscopic-assisted laser debulking and cystostomy tube placement were performed as palliative treatments, respectively, in three and one of 13 dogs that had developed UO during first-line chemotherapy. 

The presence of UO at diagnosis suggests a more aggressive tumor biologic behavior and may predispose patients to associated complications, such as UTIs [26]. Furthermore, in cases where there is no response to first-line therapy, the necessity for additional procedures arises while waiting to switch to a different chemotherapy agent. This situation can potentially discourage owners from continuing with the therapeutic protocol. This could partially account for the lower likelihood of dogs with obstruction to attain a response or sustain disease control, thereby increasing their vulnerability to tumor-related mortality.

UTIs are commonly observed in dogs with TCC, with more than 50% of dogs having at least one positive culture during the course of treatment. Additionally, one study [27] has reported that dogs with TCC in the urethra are more susceptible to UTIs when compared to dogs with tumors located in the mid-bladder region. 

Our findings revealed that dogs with a UTI at the time of diagnosis had a higher risk of experiencing local progression. TCC can disrupt the normal host barriers because of abnormal voiding patterns, compromised uroepithelium defenses, and reduced antibacterial properties due to alterations in urine pH, making the affected dogs more prone to UTI. Additionally, there is evidence suggesting that the use of antibiotics can contribute to cancer progression. In mice with tumor xenografts, the administration of antibiotics resulted in genetic alterations in cancer cells, which were associated with a reduced response to chemotherapy [28].

In addition to the prominent results derived from the statistical analysis, this study has confirmed or revealed several other intriguing findings.

Consistent with previous findings, spayed and older female dogs were over-represented in this cohort, comprising approximately two thirds of the population [2,5]. This observation further supports a potential gender predisposition for the primary urethral origin of the tumor.

According to our findings, all dogs in the study presented with chronic urologic symptoms that persisted for several weeks or months before diagnosis. The median duration detected before obtaining the diagnosis was 90 days, up to 365 days. The diagnostic delay for uTCC can be common, as initial clinical signs are often mild and may be overlooked at first [5]. Additionally, these tumors can be challenging to diagnose due to their resemblance to other conditions, such as UTI [29]. However, the duration of clinical signs did not correlate with the outcome, indicating that the severity and duration of symptoms may not necessarily be related to the stage of the disease. Notably, urethral thickening detected during digital rectal examination was observed in 51% of dogs in the cohort and may provide additional indications of tumor presence during physical examination.

In addition to the urethra, 83% of the dogs also had bladder involvement. These findings are consistent with previous studies that have shown a higher incidence of TCC occurring in multiple locations within the lower urinary tract [30]. Furthermore, two dogs showed concurrent prostate involvement. Distinguishing between prostate cancer and infiltrating high-grade urothelial cancer histologically may be challenging. However, in both cases, the histologic appearance was strongly suggestive of a urothelial origin (papillary growth, absence of glandular pattern, presence of Melamed–Wolinska bodies) [31].

The overall metastatic rate at admission was 34.4%, which is higher than previously reported [6]. Furthermore, it is quite possible that the relatively low proportion of dogs staged with TBCT in this study has led to an underestimation of this data, and more so when considering bone metastases. Based on the current findings, nodal metastasis in the iliac and inguinal lymph nodes was observed in the majority of cases, while lung involvement was reported only in two dogs. The presence of metastatic disease at the time of diagnosis did not negatively impact the outcome in the present study. This is consistent with what is reported in the literature, which states that 60% of patients with uTCC die as a result of urethral obstruction [21] along with the challenges involved in managing associated symptoms.

Chemotherapy and/or COXi represent the treatment of choice for TCC [10,11,12,13,14,15,16,17]. In this study, the administration of several chemotherapeutic drugs in combination with piroxicam resulted in a low incidence of adverse effects and an overall response rate of 28.6%. In particular, the administration of gemcitabine was effective in the control of distant progression. In human patients with metastatic TCC, gemcitabine has led to regression of metastatic lesions, supporting our observation [32,33]. 

Dogs that received radiotherapy as part of a multimodal approach were also included in the current study. RT led to partial or complete responses in three out of four (75%) dogs, indicating its potential benefit in the treatment of uTCC.

The median TSS observed in our study was 333 days. A previous study comparing two groups of dogs treated with mitoxantrone and carboplatin, respectively, reported similar survival times [3]. In another survival analysis conducted by Iwasaki, dogs with uTCC treated with or without COXi and chemotherapy had a shorter median survival time of only 121 days [1]. In our study, the use of a multimodal approach, which involved combining various treatment modalities and implementing rescue treatment with different drugs, likely contributed to the improved outcomes by aiming to achieve therapeutic synergy and delay local and distant progression.

There are weaknesses to this study that limit the extrapolability of the conclusions. First, its retrospective nature only allowed for the capture of information that was reported in the medical records. The sample size was limited, and there was lack of standardized staging and treatment protocols, which may have influenced the results of the survival analysis. 

Additionally, 63% of dogs had concurrent bladder involvement. Discriminating between bladder and urethral origin of TCC can be challenging, and we cannot be certain that all cases were primary urethral tumors. However, during the case selection process we specifically included dogs with extensive TCC urethral lesions, covering more than 75% of the urethral length, along with minor and focal involvement of the vesico-urethral junction, the trigone, and/or the prostate, as identified through a combination of endoscopy and abdominal ultrasound or CT. Due to the above, we believe that the urethra was most likely the primary site in the included cases.

It is important to acknowledge these limitations as they can affect the generalizability and reliability of the findings. Further research with larger sample sizes and standardized protocols is necessary to validate and refine the results obtained here.

## 5. Conclusions

In conclusion, this study identified UO and UTI at the time of diagnosis as negative prognostic factors in dogs with uTCC.

While no specific first-line drug was identified, our results highlight the importance of a multimodal approach for treating uTCC in dogs, with chemotherapy playing a crucial role in disease control and maintaining good quality of life. Additionally, although the sample size was small, RT showed interesting results. However, further studies are needed to evaluate the synergistic effects of different therapeutic approaches for uTCC in dogs.

## Figures and Tables

**Table 1:** Univariable Cox regression analysis of variables potentially associated with increased risk of local progression (LP), distant progression (DP), overall tumor progression (TP), and tumor-related death (TRD) in 35 dogs with uTCC undergoing medical treatment.

Variables	LP		DP		TP		TRD	
	HR (95% CI)	*p*	HR (95% CI)	*p*	HR (95% CI)	*p*	HR (95% CI)	*p*
Age > 11 ^a^	1.5 (0.7–3.4)	0.333	0.7 (0.1–3.4)	0.655	1.3 (0.6–2.8)	0.485	1.8 (0.8–4.3)	0.163
Female Sex	1.0 (0.4–2.5)	0.992	0.6 (0.3–8.1)	0.555	1.1 (0.5–2.5)	0.871	1.5 (0.6–3.6)	0.416
Neutered	0.6 (0.2–1.8)	0.370	0.4 (0.1–1.4)	0.150	0.5 (0.2–1.5)	0.220	0.6 (0.2–1.7)	0.383
Pure Breed	0.8 (0.3–1.7)	0.502	9.5 (1.2–76.6)	0.034 *	0.9 (0.5–2.0)	0.869	1.0 (0.5–2.2)	0.98
Weight > 19 kg ^a^	1.2 (0.5–2.8)	0.663	4.6 (0.9–22.3)	0.061	1.7 (0.8–3.7)	0.180	2.3 (1.0–5.4)	0.049 *
Symptom Duration > 78 Days	1.3 (0.6–3.0)	0.512	1.4 (0.3–5.4)	0.675	1.2 (0.6–2.4)	0.715	1.1 (0.5–2.5)	0.753
Azotemia at Presentation	1.6 (0.5–4.9)	0.415	2.3 (0.5–12.0)	0.310	1.8 (0.6–4.8)	0.274	1.9 (0.7–5.2)	0.228
UO at Presentation	5.9 (2.1–16.6)	0.001 *	6.0 (0.8–43.2)	0.077	4.8 (1.8–12.8)	0.002 *	5.0 (1.7–15.1)	0.004 *
UTI at Presentation	2.5 (0.9–7.1)	0.086	2.5 (0.5–12.9)	0.262	3.6 (1.4–9.0)	0.008 *	3.3 (1.3–8.5)	0.011 *
Bladder Involvement	1.3 (0.6–3.1)	0.497	0.5 (0.1–1.9)	0.324	1.4 (0.6–2.9)	0.444	1.1 (0.5–2.4)	0.824
Metastasis at Diagnosis	0.6 (0.3–1.5)	0.289	0.7 (0.2–2.7)	0.580	0.6 (0.3–1.4)	0.218	0.6 (0.3–1.4)	0.254
Medical Treatment Other Than Vinblastine	1.1 (0.5–2.6)	0.785	0.4 (0.1–1.4)	0.147	0.9 (0.4–1.9)	0.760	0.9 (0.4–2.0)	0.718
Medical Treatment Other Than Gemcitabine	0.8 (0.3–1.8)	0.571	15.2 (1.8–125.6)	0.011 *	1.2 (0.6–2.5)	0.676	1.8 (0.7–4.2)	0.196
UTI During Treatment	0.6 (0.2–1.7)	0.338	1.9 (0.5–7.0)	0.357	0.8 (0.3–1.9)	0.573	0.9 (0.4–2.2)	0.860
Rescue Treatment	1.1 (0.5–2.4)	0.916	0.5 (0.1–1.9)	0.293	0.8 (0.4–1.6)	0.481	0.7 (0.3–1.5)	0.370
Additional Procedures	3.2 (1.3–7.8)	0.013 *	2.6 (0.5–12.8)	0.243	2.3 (0.9–5.5)	0.065	1.8 (0.7–4.8)	0.214

^a^ median used as cutoff; * significant; UTI: urinary tract infection; UO: urinary obstruction.

**Table 2:** Multivariable Cox regression analysis of variables potentially associated with increased risk of local progression, overall tumor progression, and tumor-related death in 35 dogs with uTCC undergoing medical treatment.

Variables	HR (95% CI)	*p*
**Local Progression**		
UO at Presentation	5.5 (1.6–19.5)	0.008 *
Additional Procedures	1.5 (0.5–4.6)	0.455
**Distant Progression**		
Pure Breed	4.9 (0.6–41.8)	0.150
Medical Treatment Other Than Gemcitabine	9.0 (1.0–78.1)	0.047 *
**Tumor Progression**		
UO at Presentation	6.6 (2.0–22.1)	0.002 *
UTI at Presentation	6.7 (2.4–19.2)	<0.001 *
Additional Procedures	1.7 (0.6–4.8)	0.344
**Tumor-Related Death**		
Weight > 19 kg ^a^	2.5 (0.9–6.2)	0.058
UO at Presentation	3.5 (1.1–11.2)	0.036 *
UTI at Presentation	4.1 (1.5–11.3)	0.005 *

* significant; ^a^ median used as cutoff.

## Data Availability

The data that support the findings of this study are available from the corresponding author upon reasonable request.
